# Evaluation of health workforce competence in maternal and neonatal issues in public health sector of Pakistan: an Assessment of their training needs

**DOI:** 10.1186/1472-6963-10-319

**Published:** 2010-11-27

**Authors:** Shabina Ariff, Sajid B Soofi, Kamran Sadiq, Asher B Feroze, Shuaib Khan, Sadiqua N Jafarey, Nabeela Ali, Zulfiqar A Bhutta

**Affiliations:** 1 Department of Paediatrics & Child Health, Aga Khan University, Karachi, Pakistan; 2 PAIMAN, Pakistan Initiative for Maternal and Neonatal Health, JSI Research & Training Inc, Islamabad, Pakistan; 3 Department of Obstetrics and Gynaecology, Ziauddin Medical University, Karachi, Pakistan

## Abstract

**Background:**

More than 450 newborns die every hour worldwide, before they reach the age of four weeks (neonatal period) and over 500,000 women die from complications related to childbirth. The major direct causes of neonatal death are infections (36%), Prematurity (28%) and Asphyxia (23%). Pakistan has one of the highest perinatal and neonatal mortality rates in the region and contributes significantly to global neonatal mortality. The high mortality rates are partially attributable to scarcity of trained skilled birth attendants and paucity of resources. Empowerment of health care providers with adequate knowledge and skills can serve as instrument of change.

**Methods:**

We carried out training needs assessment analysis in the public health sector of Pakistan to recognize gaps in the processes and quality of MNCH care provided. An assessment of Knowledge, Attitude, and Practices of Health Care Providers on key aspects was evaluated through a standardized pragmatic approach. Meticulously designed tools were tested on three tiers of health care personnel providing MNCH in the community and across the public health care system. The Lady Health Workers (LHWs) form the first tier of trained cadre that provides MNCH at primary care level (BHU) and in the community. The Lady Health Visitor (LHVs), Nurses, midwives) cadre follow next and provide facility based MNCH care at secondary and tertiary level (RHCs, Taluka/Tehsil, and DHQ Hospitals). The physician/doctor is the specialized cadre that forms the third tier of health care providers positioned in secondary and tertiary care hospitals (Taluka/Tehsil and DHQ Hospitals). The evaluation tools were designed to provide quantitative estimates across various domains of knowledge and skills. A priori thresholds were established for performance rating.

**Results:**

The performance of LHWs in knowledge of MNCH was good with 30% scoring more than 70%. The Medical officers (MOs), in comparison, performed poorly in their knowledge of MNCH with only 6% scoring more than 70%. All three cadres of health care providers performed poorly in the resuscitation skill and only 50% were able to demonstrate steps of immediate newborn care. The MOs performed far better in counselling skills compare to the LHWs. Only 50 per cent of LHWs could secure competency scale in this critical component of skills assessment.

**Conclusions:**

All three cadres of health care providers performed well below competency levels for MNCH knowledge and skills. Standardized training and counselling modules, tailored to the needs and resources at district level need to be developed and implemented. This evaluation highlighted the need for periodic assessment of health worker training and skills to address gaps and develop targeted continuing education modules. To achieve MDG4 and 5 goals, it is imperative that such deficiencies are identified and addressed.

## Background

### Situational Analysis

Each year almost 8.8 million children aged less than 5 years die [1]. Of these 3.71 million deaths, occur during the first 28 days of life and another 2.3 million in the immediate post neonatal period [2]. About 12% of neonatal deaths are due to prematurity, 9%to birth asphyxia, 6% due to sepsis and 4% to pneumonia [1]. Pakistan is amongst the five countries that contribute 49% of all childhood deaths. Each day almost 500 newborns die in Pakistan and an estimated 216,000 die before they reach the age of one month. The state of maternal health is an even greater challenge, with a mortality ratio of 276 deaths per 100,000 live births [2,3]. One in every 89 Pakistani women, dies due to causes related to childbirth as compared to 1 in 8,000 in the developed world [2].

Maternal and Child Health are deeply intertwined and initiatives to improve health of mothers also benefit newborns and children, thereby creating the foundation for a healthy life [4]. Despite a large number of vertical child health survival programmes, there has been little or no improvement in perinatal and neonatal health and the rates of reduction in under 5 mortality are among the slowest in the region [3]. Pakistan has the eighth highest rate of neonatal death, ranking only below Afghanistan and Iraq amongst Asian countries [3].

Despite having one of the best Public Health infrastructures in Asia, Pakistan's poor health statistics point out a glaring lack of quality in care dispensed, and, implementation of 'standard' principles. The scarcity of skilled health personnel is a major contributory factor [5].

Approximately 20-21% of neonatal deaths and 29 -40% of all post neonatal deaths in children less than 5 years can be prevented by simple, evidence-based practices such as exclusive breastfeeding, prevention of hypothermia and infection and promotion of hand washing [1,6,7]. Implementing robust training programmes and developing appropriately targeted curricula for healthcare professionals brings a marked improvement in their knowledge and skills which, in turn, translates into improved healthcare outcomes [8,9]. But for such initiatives to effectively realize their full potential, it is essential to accurately assess training needs and consider other contextual factors that could influence the outcome of these programmes [10,11].

To design a needs specific, district-level training programme for healthcare providers in the public health sector we carried out training needs assessment (TNA) exercise. This was an initiative conducted under the umbrella of PAIMAN, 'Pakistan Initiative for Maternal and Newborn Care' funded by USAID. The Department of Paediatrics & Child Health, Aga Khan University, Karachi in close collaboration with National Committee on Maternal & Neonatal Health (NCMNH) developed the TNA tool with the concept of continuum of care for mothers and newborns.

## Methods

The objective was to assess training needs of health care providers in the public sector and accurately identify gaps in knowledge, skills and attitudes in MNCH knowledge and skills.

### Settings

Pakistan, with an estimated population of 185 million people is strategically located in South Asia with the Arabian Sea to its south, India to the east, Iran and Afghanistan to the west and China to the north. The country has four provinces: Sindh, Punjab, Baluchistan and the North-West Frontier Province. Each province is further subdivided into administrative functional districts.

**Figure 1:**
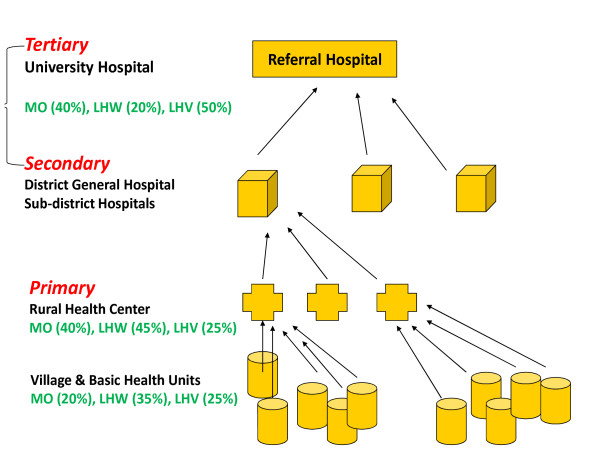
**Depicting the public health infrastructure in Pakistan and break up of health care providers in percentages**.

Pakistan's public health delivery system functions as part of an integrated health infrastructure with a hierarchal organization (Figure 1). Majority of healthcare providers (LHW, LHV, midwives, nurses and doctors/medical officers) are employed in the public health sector that is centralized under the federal and provincial ministries of health [12,13].

### Study Population

All three cadres of health care providers (LHWs, LHVs, /midwives/nurses, and doctors) undertook the training needs assessment. Presently, there are 100,000 LHWs employed in the community[14]. The LHWs are females with a minimum of eight years of education, residents of the locality in which they are working. They functions primarily as community health worker (CHW) providing an essential link between the formal health system and the communities. Following recruitment, they undergo 15 months of preparation. This includes three months of classroom sessions, followed by supervised field training for 12 months.

The LHW maintains a death registry, provides health education on hygiene and sanitation along with motivation and referral services for safe motherhood (family planning, antenatal care and safe delivery).They also conduct one postnatal visit, and provides health education on immediate newborn care. Although trained to provide basic neonatal resuscitation with mouth-to-mouth breathing, at present, they do not attend deliveries.

Midwives, LHVs and nurses constitute another important cadre and are usually the first point of care during the reproductive health period. Presently, there are 7073 LHVs, 23897 midwives, and 33427 nurses in the country[15,16]. The LHV undergoes a 2-year training program that comprises one year of midwifery and a second year in paediatrics and tropical diseases. She conducts deliveries at household and facility level, and provides immediate newborn care. The midwifery-training is an 18-month program and consists of classroom sessions and clinical attachments in maternity units[13].

The physician cadre, known as Medical Officer (MO), in the public sector is the most qualified in this multilevel set up. In Pakistan, the minimum qualification to enter a medical school is 12 years of schooling with last two years of education in compulsory science subjects (biology, chemistry and physics). The medical education is a 5 years training program, followed by 1 year of mandatory supervised practical training (internship).

### Description of Health Care Facilities

The Basic Health Unit (BHU) is a primary care centre with provision of preventive and curative services including antenatal care, immunization and family planning. The Rural Health Centre (RHC) functions as a referral unit for BHU.Tehsil/Taluka and District Hospitals are in turn, referral centres for RHCs. Essential and emergency obstetric and neonatal care is provided at RHC, Tehsil/Taluka and District Hospitals with varying levels of quality and consistency.

(Table 1)

**Table 1:** Organization of National Health Care System in Pakistan

Facility	Population Served	Functions	Care Providers
**Dispensary**	5000	Outpatient curative services only	1 MO, 2 dispensers,3 TBA, 4 others

**Maternal and Child Health Centre (MCH)**	20,000	MCH services; midwifery services, such as deliveries; not included: obstetric emergencies	1 LHV, 1TBA, 1 community health work

**Basic Health Unit (BHU)**	10,000	Basic services including MCH and FP, but no labor rooms	1 MO, 1 LHV, 1 dispenser, 1TBA, 1 dresser

**Rural Health Centre (RHU)**	50,000 - 100,000	Referral center for BHU; inpatient services including 10-12 beds; ambulance services available; obstetric emergency services and lab services	3 MOs (2 M,1 F),1 nurse(position unoccupied); 2LHVs, nursing assistant, dental technician, vaccinator, technical staff for labs.

**Tehsil Hospital (sub-District Hospital)**	380,000	In-patient services including 30-80 beds; obstetric emergencies services	6 MOs, 1 surgeon, 1 pediatrician, 1 gynecologist, 1 dental supervisor, 10 nurses, 1 LHV, 5 dispensers, 4 technicians, 35 others

**District Headquarters Hospitals**	1.16 million	In-patient services including 100-300 beds	21 MOs, specialist, including surgeon, peiatrician, gynecologist, anesthesist, dermatologist,17 nurses,23 paramedics,67 others

**Teaching Hospitals and Ministry**	City -based	Tertiary care services	Include all types of medical, nursing and other categories of health care providers

F = Female; FP = Family Planning; LHV = Lady Health Visitor; M = Male; MO = Medical Officer; TBA = Trained Birth Attendant.

**Source: **Memon RJR. Implementation problems of maternal health policies and program. Provincial seminar for maternal health. Karachi, Pakistan, 1997

### TNA Tools

Tools were designed to integrate maternal and neonatal components into a single tool adapting to a life-cycle approach. We selected clinical areas in MNCH that directly or indirectly contribute to maternal and newborn morbidity, mortality, and interventions that potentially prevent adverse outcomes. These were adopted from evidence published in Lancet series on Maternal and Newborn Child Survival Series [17-19].

Knowledge was assessed on breastfeeding, immediate postnatal care, management of newborn infections, care of low birth weight babies and [5] newborn resuscitation. Similarly, key areas on maternal care included antenatal visits, postpartum care, third-stage labour management, emergency obstetric practices and reproductive health [20].

Tools for each cadre of health care provider were unique, but a degree of uniformity retained. All the three tools on knowledge, skills and attitude were quantitative in design.

### Sample Size

The TNA was a novel exercise of health care providers across the country with no preceding information on their competency. We thus assumed base line knowledge and skills of health care providers to be 50% and desired level of absolute precision as +/-5% with level of confidence interval 95% and used the following formulas for sample size estimation[21]

$$\text{n}=\frac{Z_{1-\alpha /s}^{2}\;\text{P}(1-\text{P})}{{\text{d}}^{2}}$$

Where:

**P **anticipated value of proportion assumed 50%

**d **desired level of absolute precision **+/-**5%

**α **is level of significance taken 95%

The sample size thus obtained was 370. Based on this, 370 health care providers were required from 10 selected districts of PAIMAN. We selected 30 health care providers from each district with representation from each cadre. (Table 2)

**Table 2:** Number of Participants & Marks allocated to the TNA Tool

Cadres	Knowledge		Resuscitation skills		Counselling skills	
	**Number of Participants**	**Marks** **Allocated**	**Number of Participants**	**Marks** **Allocated**	**Number of Participants**	**Marks** **Allocated**

**Medical officers**	94	87	95	30	94	37

**Lady health visitors**	172	87	170	20	172	40

**Lady health workers**	98	62	98	15	98	40

### Evaluation of Methodology for Knowledge and Skills

Knowledge was assessed by means of semi-structured, close-ended questions (MCQs) while skills evaluated by observing the performance of healthcare providers in newborn resuscitation. Resuscitation skills were assessed using a standardized checklist adapted from the neonatal resuscitation program (NRP) guidelines by American heart association and American Academy of Pediatrics (5^th ^Edition.2006).Competency was evaluated on demonstration of resuscitation steps on a mannequin. Skills assessment for LHWs/LHVs/nurses was limited to mouth-to-mouth breathing. An experienced paediatrician with certification in newborn resuscitation skills simulated a uniform resuscitation scenario.

Attitudes of healthcare providers were assessed by evaluating their counselling skills.

### Data Collection Processing and Analysis

To calculate the total score of a participant we divided their earned points with the total number of points allocated for the exercise. The knowledge assessment tool for doctors allowed a maximum of 87 marks and for LHWs 62 marks. Further, we divided the results (in percentage) for each cadre to create uniformity and for comparisons. The results were stratified based on percentage--with 0- 10 per cent being the lowest, and 91-100 per cent the highest. We replicated the same for all three cadres for both the maternal and neonatal components. We also calculated the mean score (in percentages). Data was entered using FoxPro and analyzed by SPSS (version 13).

### Competency Level

We defined competency as 'having sufficient knowledge and skills to comply with predefined clinical standards'. We defined an arbitrary competency level for each individual tool. Since cross-country comparisons were a key component of the exercise, we used international standards[22,23].

### Ethical Approval

Ethical approval for the study was obtained from the National Research Ethics committee and verbal consent sought from the participating health care providers prior to the assessments.

## Results

### Outcomes in the Knowledge Assessment

The knowledge component was attempted and completed by (n = 364) primary healthcare providers (98% the total sample size). The remaining six participants reported absent.

**Figure 2:**
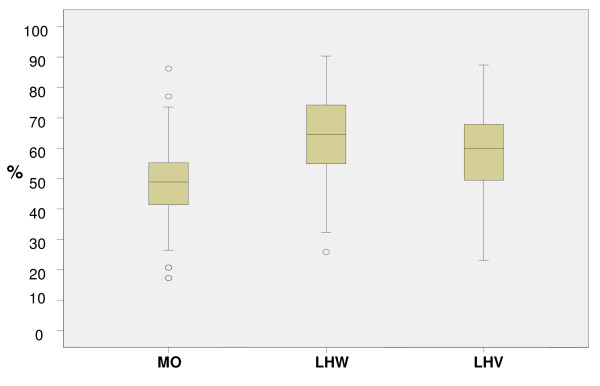
**Outcomes in the Knowledge Assessment Tool (Maternal and Newborn) showing median with inter-quartile range**.

The medical officers performed poorly as compared to other cadres Only 6 per cent (5/94) of the MOs achieved > 70 per cent of the total allocated marks while 9.5 per cent (9/94) scored between 61-70 per cent. Approximately 30 per cent scored 50 per cent of the total allocated marks. A significant number (34/94 or 36.2 per cent) achieved a score less than minimum competency level of 50%. (Figure 2) depicts the performance of all three cadres in knowledge assessments.

**Figure 3:**
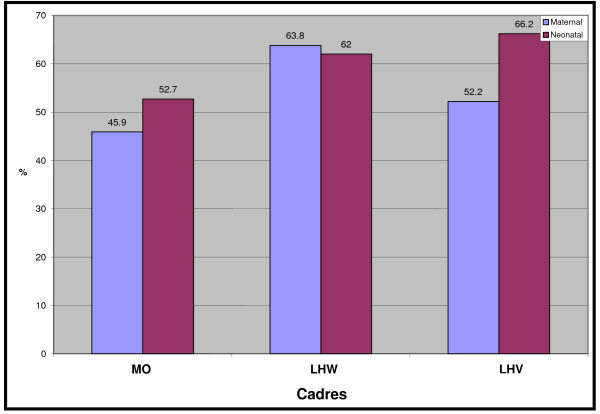
**Comparison of Average Score Obtained by Different Cadres in Maternal & Neonatal Knowledge Tools in percentage**.

The mean percentage scores of MOs in the maternal component was 45.9 (S.D ± 11.9) and for neonatal 52.7 (S.D ± 19.1). Mean scores of MOs in knowledge at the district level varied. The MOs working in district Lasbella had the least mean score of 33.7 (S.D ± 9.6) and Jhelum the highest of 61.0 (SD ± 10.5). The performance of MOs on knowledge related to recognition and management of newborn sepsis was comparatively poor to LHWs and LHVs (with 'P' values of 0.004 and 0.000). (Figure 3) describes the comparative scores in maternal and neonatal knowledge assessments in all three cadres.

The LHVs had better knowledge of birth preparedness and newborn resuscitation as compared to MOs (70.5 per cent versus 54.3 per cent). Both the MOs and LHVs cadre had poor knowledge of active management of third-stage of labour. Most participants answered that they would use Ergometrine rather than Oxytocin to initiate active management of third-stage of labour. All three cadres had sufficient knowledge of the importance of antenatal visits and scored above 70 per cent.

### Outcomes in Skills Assessment

**Figure 4:**
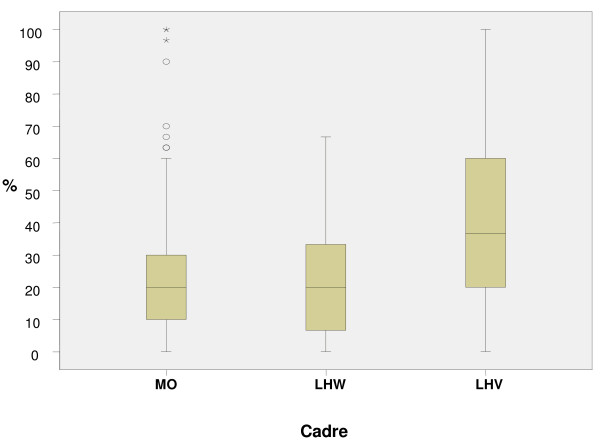
**Outcomes in the Skill based Resuscitation showing median with inter-quartile range**.

Skills were assessed by observing (1) immediate care of newborn and (2) neonatal resuscitation, with both procedures performed on anatomical models. Only 33 per cent (123/370) of the participants attempted the skills assessment. Of these, 50 per cent were LHWs, 32 per cent MOs and the remaining were LHVs/nurses/midwives. All three cadres scored poorly, especially the MOs and LHWs. (Figure 4) shows the median with inter-quartile range for resuscitation skills for all three cadres.

We observed certain lapses in the immediate newborn care skills assessments that could be critical for the survival of a newborn. Majority (54%) of all health care providers failed to check the newborns respiration and provide adequate thermal protection. Most MOs (78 per cent) were unable to carry out the step of drying the newborn. Only 45 per cent assessed the breathing and heart rate.

In the assessment of resuscitation skills, merely 22 per cent participants correctly performed steps to prepare an infant for resuscitation. Only 36 percent of the MOs cadre correctly placed the newborn on a dry surface, and 27 per cent were able to maintain an airway.

Relatively higher proportion (68%) cleared the air passage with suction and 35 per cent could provide effective ventilation through an ambu bag and mask. Only 32 per cent of MOs identified the landmark for cardiac compression and more than 80 per cent were unable to carry out the steps of intubation. Merely 23 per cent administered epinephrine during the resuscitation process.

In addition to basic clinical ability and appropriate levels of knowledge, effective interpersonal communication skills are vital for successfully delivering healthcare [14]. In this area of proficiency, the MOs cadre from district Bunner scored the highest with mean scores of 63.7 while the MOs' of district Khanewal scored the lowest with mean score of 35.7. Only 41 LHWs out of 98 participated in the counselling skills assessment and completed the session. The remaining 57 were hesitant and requested to abstain. Of those who participated in the exercise, only 50 per cent managed to score half the allocated marks. (Table 3)

**Table 3:** Mean scores in percentage obtained by different cadres on Counselling Skills

Cadres		Total number of participants (n)	Mean scores in %	SD ±
**Counselling** **Skills**	**RMO**	94	47.4	± 13.5

	**LHW**	98	32.9	± 9.7

	**LHV**	172	34.8	± 10.6

### Relative Competencies in Maternal and Neonatal Components

The MOs performance in knowledge and skills was better in the neonatal component as compared to maternal. Conversely, the performance of LHWs in maternal care was relatively better than in the neonatal care (63.8 per cent versus 62.0 per cent). The MOs demonstrated better competency in the neonatal component of MNCH as compared to maternal component.

The LHWs competencies in maternal component were marginally better than in the neonatal component (63.8 per cent versus 62.0 per cent).

## Discussion

The overall results must be considered poor, in particular those of the (MOs) cadre taking into consideration their extensive academic training. We assumed the (MOs) to obtain a minimum competency level, fixed at an arbitrary 50% cut off in the knowledge assessment of MNCH. Despite the fact that the tools were designed to assess just basic level of knowledge, only 30 per cent of MOs cadre managed to score above the minimum competency level. This indicates a serious deficiency in knowledge of basic maternal and neonatal care.

Our findings reflect the country's ailing health system and emphasize the view that very few medical institutes currently provide optimal training on essential maternal and newborn health issues. Doctors in the public health sector do not receive any formal training or refresher courses after they graduate from medical school (MBBS) which further compounds their lack of competency. Resultantly, these doctors remain uninformed and professionally insecure in a poorly administered public health system, especially in the underdeveloped and remote parts of the country.

It is worth noting the intra cadre and inter cadre scores variation in resuscitation. For the doctor's this could be due to lack of exposure to neonatal resuscitation. Although medical schools have a uniform curricula, internship in specialities may differ. Therefore, a graduate who rotates in ophthalmology and Internal medicine would never have a chance of coming across an obstetric or neonatal outpatient let alone an emergency. After internship; graduates employed by the public health sector spend compulsory allocated time at BHU/RHCs. This leaves young doctors unsupervised and unprepared to deal with the realities of resource-poor settings in the communities, leading to absenteeism and failure to provide quality MNCH care.

In the public sector, there is also a lack of sincere will and effort on the government's part to improve the overall status of health facility and health care providers. Only 0.57percentage of GDP is spent on health. This is less than what Bangladesh and Srilanka spends on health budget [24]. The health facility assessment carried out under PAIMAN reflected disparity among districts in provision of health care facilities (including trained personnel, medicine, equipment and ancillary services). Insufficient and inconsistent resource allocation is one of the major factors among many, contributing to the disparity.

The findings of our knowledge assessment are indicative of a serious deficiency in the public healthcare system's capacity to make a serious indent in the maternal and newborn mortality rates of the country and thus a matter of concern. Our findings were similar to needs assessment exercise on MNCH care conducted in Vietnam and Nairobi [25,26].

It is imperative that primary healthcare providers (LHWs, LHVs, doctors, midwives) have sufficient obstetric and resuscitation skills if the outcomes of maternal and neonatal health are to be improved [12,27-29].

The relatively better resuscitation skills of (LHVs/midwives/nurses) in comparison to MOs and LHWs are perhaps a reflection of greater exposure to childbirths and immediate newborn care. Given that birth asphyxia contributes 23% of the perinatal mortality in Pakistan, competency in resuscitation skills, should be considered a minimum requirement for all health care providers, irrespective of cadre. The disappointing performance of MOs and LHWs in this essential skill, underscores the urgent need for intensified training. In fact, resuscitation skills are so critical to neonatal survivability that these need evaluations periodically.

In contrast to the MOs, the LHWs performed comparatively better in knowledge assessment despite having limited formal medical education [30]. This must be viewed positively as it gives rise to the possibility of effectively employing LHWs in implementing efficient community outreach programmes [29].

The performance of the LHWs in counselling was the poorest amongst all three cadres highlighting communications skills as the most important training needs of this cadre. This reflects on the inherent reluctance of LHWs to discuss sensitive issues like family planning and reproductive health due to social taboos especially when most of them are young girls. This may also be due to lack of practical training during formal training course. Considering the fact that LHWs are the first interface between communities and the health system, importance of optimal counselling cannot be undermined. In a recent survey carried out on the community health workers own perception of knowledge and communication skills revealed similar findings [14].

The poor performance of healthcare providers of Baluchistan is attributable to their suboptimal working conditions, poor infrastructure and lack of even basic functional equipment needed for childbirth. Lack of resources result in negative healthcare consequences; it is known that the poor segment of a population benefits less from public spending in health and that inequity is a complex and common problem in all developing countries including Pakistan [18,19,31,32].

### Limitations of the TNA Exercise

Our study had few limitations. We were cognizant that the sampling size and frame could not be representative of all ten districts it would yet provide an illustrative model. The comparison of assessments among a non-homogenous group of healthcare providers may appear unfair, but these groups were different tiers of same health profession and system. We made a conscious effort to ensure levels of assessments were concurrent and aligned with the curricula and competency, while maintaining uniformity in clinical areas assessed.

For instance, we tested skills of all three cadres in resuscitation, for MOs, the competency check list included intubation and administration of drugs. But for LHWs and LHVs initial basic resuscitation steps with mouth to mouth breathing were assessed. We therefore considered worth carrying out a comparison among the cadres.

Questions were based on recommendations in World Health Organization (WHO) guidelines on Integrated Management of Childhood Illnesses IMCI. However we faced the challenge of defining competency levels as there were no national benchmarks.

We elected to use MCQs because we believed this was the most effective way to assess knowledge [33]. There were added barriers of language and competency of the participants in comprehending the knowledge based tool. The non-availability of gender breakup of the participants and its influence on assessment outcome, especially in the maternal component, was also a matter of concern.

The accepted competency level for each tool was neither scientifically driven nor uniform. We felt this was a significant limitation. However, without setting up an arbitrarily baseline competency level, the assessments outcomes was not possible.

## Conclusion

There is a vital need to improve current knowledge and skills through continuing medical education and relevant training imparted to all cadres of healthcare providers in fundamental maternal and newborn problems. We are of the view that this improvement in knowledge and skills will translate into improved maternal and neonatal health outcomes.

We may be able to significantly reduce the number of neonatal deaths caused by asphyxiation, and lessen the incidence of disabilities resulting from birth asphyxia by simply enhancing basic resuscitation skills [6,18,19,34].

Along with improvement in the curricula and training it is imperative to review the methodology of training and develop means of continuous medical education. Perhaps a modified form of educational program, using best evidence teaching methods with emphasis on practical hands on training program could be implemented. This program may consequently result in significant improvement in maternal and neonatal mortality [8,10].

Finally, counselling skills of all cadres of the healthcare staff receive little importance during the training period. The results of TNA aptly reflect the deficiency in this important skills component. Counselling being a critical component of problem management, it should receive due attention by the relevant authorities. Special emphasis is needed in undergraduate and postgraduate curricula and training. Similarly, communication skills modules need to be included in all relevant training courses of health care providers.

## Key Message

To achieve the MDG 4&5 a reduction in perinatal mortality is imperative. A concerted effort by all stakeholders is required to revise current curricula and training guidelines. Periodic quality checks and refreshers are also required, followed by redressal exercises to optimize system reform.

## Competing interests

The authors declare that they have no competing interests.

## Authors' contributions

ZAB, conceptualized the study, SA, SBS, KS and SNJ developed the TNA tool. NA & SK provided technical support (PAIMAN), AF & SBS supervised the data collection, sifted through the data and performed the statistical analyses. SA was the lead author supervised by ZAB.

All authors read and approved the final manuscript.

## Pre-publication history

The pre-publication history for this paper can be accessed here:

http://www.biomedcentral.com/1472-6963/10/319/prepub
